# Mammographic and Ultrasonographic Findings of Different Breast Adenosis Lesions

**DOI:** 10.5334/jbr-btr.850

**Published:** 2015-09-15

**Authors:** E. Ozturk, C. Yucesoy, B. Onal, U. Han, G. Seker, B. Hekimoglu

**Affiliations:** 1 Department of Radiology, Ankara Diskapi Training and Research Hospital, Ankara, Turkey; 2 Department of Pathology & Cytology, Ankara Diskapi Training and Research Hospital, Ankara, Turkey; 3 Department of Surgery, Ankara Diskapi Training and Research Hospital, Ankara, Turkey

**Keywords:** Breast, adenosis, ultrasonography, mammography

## Abstract

**Aim:** To describe imaging features of different breast adenosis lesions.

**Materials and methods:** Mammographic and ultrasonographic findings of patients with different types of adenosis were reviewed retrospectively Tissue samples were obtained either with US-guided core needle biopsy or localization with needle-wire system and surgical excision.

**Results:** Forty-three adenosis lesions were diagnosed in 41 patients: 27 sclerosing adenosis, 13 blunt duct adenosis and 3 microglandular adenosis. Most frequent abnormal findings of sclerosing adenosis were masses with non-circumscribed margins and focal acoustic shadowing without mass configuration (54%) on ultrasonography. Mammography was normal in 54% of sclerosing adenosis, the most common abnormality was architectural distortion (21%). In blunt duct adenosis, usually circumscribed masses (46%) were detected on ultrasonography, clustered punctate microcalcifications (23%) and circumscribed masses (23%) were observed on mammography. All microglandular adenosis lesions were non-circumscribed masses. Premalignant components were detected only with surgical excisional biopsy in three patients that showed suspicious radiological findings and benign pathological result on core biopsy.

**Conclusion:** The adenosis lesions have no pathognomonic characteristics on mammography and ultrasound. Total excision may be considered when suspicious radiological findings are present although core needle biopsy results are benign.

Adenosis refers to histological hyperplasia that primarily involves the glandular component of the breast. This lesion corresponds to an enlargement in size of the lobule and terminal ductal lobular unit, characterized by an increased numbers of ductules and acini within the lobule [1,2]. Different types of adenosis were defined such as sclerosing adenosis (SA), blunt duct adenosis (BDA), microglandular adenosis (MGA), apocrine and adenomyoepithelial adenosis [3]. The radiological features of adenosis have been reported in a small number of articles published in the imaging literature [4,5,6].

Breast adenosis can be misinterpreted as breast cancer radiologically [4,5]. Core needle biopsies have to be adequate to avoid overlooking possible malignant alterations [7,8]. The well-known form of adenosis is SA, mainly because of the high likelihood of being misdiagnosed as carcinoma on fine needle or core biopsy [9]. However other types of adenosis can have important clinical concerns, nearly one third of cases of microglandular adenosis may harbour an invasive carcinoma [3]. It is important to be aware of the radiological features of adenosis to minimize unnecessary diagnostic procedures and to prevent inappropriate management.

We present the mammographic and ultrasonographic findings of different adenosis lesions with their managements.

## Material and methods

Study was approved by the institutional ethical review board. Medical records of 1386 patients who underwent a breast biopsy (14 G core needle biopsy or excisional biopsy) between 2004 and 2013 in our unit were reviewed. Lesions in which adenosis constituted more than 50% of the epithelial component histopathologically were included in this study.

Mammography was performed by the use of Fischer Imaging – HFX Plus (HFX Plus – Fischer Imaging, Denver, CO., USA) with two routine positions (craniocaudal and mediolateral oblique). Sonographic examinations were performed with Toshiba, Power Vision 6000 SSA-370A (Tokyo, Japan) using 6–11 mHz high frequency linear transducer or with GE Logiq S6 (GE Healthcare, Milwaukee, Wisc., USA) 7–12 mHz broadband transducer. The mammograms and ultrasonographic examinations were reviewed in consensus by two experienced radiologists who were specialized on breast imaging for more than 10 years. Lesions were classified according to the BIRADS (Breast Imaging Reporting and Data System) [10].

Fourteen non-palpable lesions were sampled via 14G core needle biopsy (Bard Max-Core 14 G × 10 cm, Bard Biopsy Systems, USA) with sonographic guidance. Seven of these had undergone surgical excisional biopsy due to presence of suspicious radiological findings but core needle biopsy results were benign. Nineteen non-palpable lesions were localized with needle-wire system under sonographic guidance and two palpable lesions had undergone total surgical excision due to patient preference. Eight lesions which were detectable only on mammography were localized using a needle-wire system and surgical excision was performed. Complete removal of all lesions that evaluated with excisional biopsy was confirmed with specimen radiographs or US.

## Results

Forty three breast adenosis lesions were identified in 41 patients. Thirty-eight patients were examined by mammography and ultrasonography (US); three patients who were younger than 30 years old were examined only by US. All patients were female, aged between 20 and 78 years (mean 44.7 years). Two (4.9%) patients had a history of breast cancer in the contralateral breast and another five (12%) cases had family history of breast cancer. Nine (22%) patients presented with palpable masses, mastalgia was the presenting complaint in fourteen (34%) patients. For eighteen patients (44%), the adenosis lesion was detected by screening. The mean lesion diameter was 14.3 mm (range: 7–32 mm). The histopathological examination results were as follows: 27 lesions were described as SA, 13 lesions were diagnosed as BDA and 3 were classified as MGA. SA was accompanied with atypical epithelial hyperplasia in 3 lesions and ductal carcinoma in situ (DCIS) in 1 lesion. DCIS associated with SA was not detected on core biopsy, likewise the 2 lesions with atypical hyperplasia. They were diagnosed only with surgical excisional biopsy.

### Mammographic and ultrasonographic findings of adenosis subtypes

**Figure 1:**
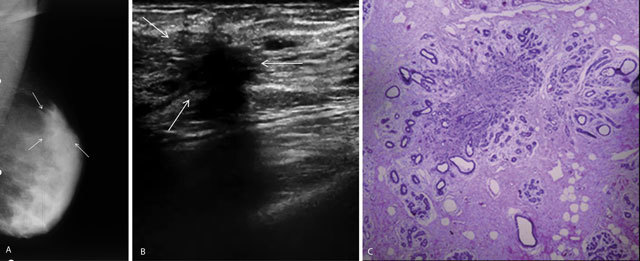
A 42-year-old woman with mastalgia. A. Left mediolateraloblique mammography shows architectural distortion (arrows). B. Ultrasonography shows a spiculated mass with acoustic shadowing (arrows). C. Histopathological diagnosis was sclerosing adenosis (c) (H&E × 20); enlarged lobule, distorted by scar-like fibrous tissue and having proliferating cells of myoepithelial nature in the center and enlarged ducts at the periphery.

Sclerosing adenosis: twenty-seven lesions were detected in 26 patients. The mammograms showed five (21%) focal architectural distortion, three (12%) circumscribed masses, two (8%) clustered microcalcifications, one (4%) spiculated mass and one (4%) asymmetrical focal density combined with macrocalcification. The mammograms were in normal limits in 13 (54%) patients. Ultrasonographic examination revealed 4 lesions of focal acoustic shadowing without mass configuration (18%). Eighteen masses were detected in 17 patients on US. Characteristics of these masses were as follows; eight were (36%) circumscribed (all were diagnosed as nodular adenosis on biopsy), five were (23%) microlobulated (2 cases were reported as nodular adenosis histologically), four were (18%) indistinctly marginated and one was (5%) spiculated (Fig. 1).

**Figure 2:**
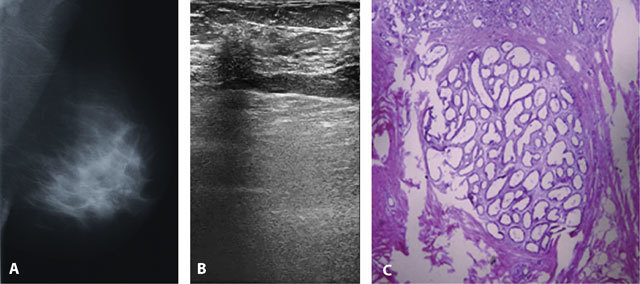
A 45-year-old woman who presented with mastalgia. A. The left mediolateraloblique mammography was negative with dense breast composition. B. Focal acoustic shadowing without a mass configuration was seen on ultrasonography. C. Histopathological diagnosis was blunt duct adenosis (H&E × 40); the breast lobule displayed blunting of both the lateral outlines and the tips of the ducts that are lined by two cell types.

Blunt duct adenosis; Thirteen BDA were detected in 12 patients. These lesions were detected thanks to a clusters of punctate microcalcifications (n = 3) or a mass (n = 10). All the masses (n = 3) detected by mammography and US were with well-circumscribed margins. Seven masses were seen only on US: 3 with circumscribed margins, 3 with non-circumscribed margins and 1 (8%) with a focal acoustic shadowing (Fig. 2).

**Figure 3:**
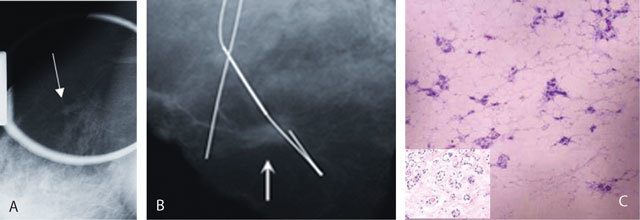
A 45-year-old woman with left mastectomy. A, B. Spot compression mammography on right mediolateraloblique position and specimen radiography showed spiculated mass (arrows). C. Histopathological diagnosis was microglandular adenosis (H&E × 40); haphazardly distributed small round glands with eosinophilic luminal secretion and lacking myoepithelial cells (inset × 100) are seen.

Microglandular adenosis; three MGA lesions were observed as masses, all (100%) with non-circumscribed margins. A spiculated mass was detected on both mammography and ultrasonography (Fig. 3). Other two lesions were observed only on US as a hyperechoic mass with indistinct margins and a microlobulated mass.

The correlations of mammographic and ultrasonographic findings with histologically results are summarized in Table 1.

**Table 1:** Correlation of mammographic and ultrasonographic findings with histologically results.

Mammography	Ultrasonography														
		
		Sclerosing adenosis						Blunt duct adenosis						Microglandular adenosis					
		
			masses						masses					masses		
								
		Negative	circumscrbed	Microlobulated	Indistinct marginated	spiculated	focal acoustic shadowing without mass configuration	Negative	circumscrbed	Microlobulated	Indistinct marginated	spiculated	focal acoustic shadowing without mass configuration	Microlobulated	Indistinct marginated	spiculated
Normal			5	4	1	1	2		2	1	2		1	1	1	
Masses	circumscrbed	1	2						3							
	spiculated				1											1
Architecural distortion		1			2		2									
Clustered microcalcifications		2						3								
Focal asimetrical density		1														
Did not performed (age < 40)			1	1					1							

## Discussion

Adenosis refers to benign proliferative process that affects mainly the lobular (acinar) component of the breast parenchyma [3]. Although it is a benign disorder, it is considered as an important entity due to mimicking malignancy both clinically and histologically. Adenosis was described as SA, blunt duct adenosis (BDA), microglandular adenosis (MGA), apocrine and adenomyoepithelial adenosis [3].

Sclerosing adenosis is the most common type of adenosis. It is described as the proliferative lesion of the terminal duct lobular unit and characterized by an increased number of acini that may either produce a mass (adenosis tumor, nodular adenosis or nodular sclerosing adenosis) or become surrounded by stromal sclerosis (sclerosing adenosis) [1,6]. It can manifest as a clinically palpable mass or as a suspicious finding on mammography or on ultrasonography [1,2,4,11,12].

Published studies about the imaging of characteristics of nodular adenosis are very limited in number [6,13]. According to Dipiro et al. 70% of nodular adenosis were circumscribed noncalcified masses on mammography and oval masses on US [6]. On the other hand, Neilsen and Nielsen stated that adenosis tumors appear mostly as irregular density on mammography [13]. In our series most (80%) of the nodular adenosis lesions were well-circumscribed and oval-hypoechoic masses, while 20% of lesions had microlobulated margins on US. However the mammography was mostly (80%) in normal limits in nodular adenosis patients and only two (20%) nodular adenosis lesions were observed as circumscribed masses on mammography. A focus of SA may be associated with spiculated lesion or nodules with indistinct margins or asymmetrical focal density [5,14]. Although radiolucent centered spiculated lesions was reported to suggest radial scar or SA compared to the usually opaque centered carcinoma, it is often impossible to differentiate such an area from malignancy with certainty on mammography [14,15]. SA lesions may also display opaque centers. Opacity or radiolucency of the lesion center depends on histological morphology; cellularity, sclerosis and fat content [5]. In our series, five architectural distortion, one spiculated mass and three circumscribed masses were observed on mammography. Three of five (60%) lesion with architectural distortion had radiolucent center. SA was reported as asymmetrical focal densities in so few (nearly 7%) cases by Taskin et al and Bilgen et al [4,5]. Similarly we observed asymmetrical focal density with indistinct margins and macrocalcifications in only 1 patient (4%).

Microcalcifications in SA may be observed in numerous forms such as clustered, amorphous or indistinct, pleomorphic or scattered punctuate [4,12]. Bilgen et al reported clustered punctate or irregular microcalcifications as the most common (55.8%) findings of SA [5]. However Taskın et al and Gill et al observed clustered amorphous or pleomorphic microcalcifications with rates of 39% and 45.5% respectively and masses were detected more frequently than microcalcifications in their series [4,12]. Similarly masses (70%) were seen more often than microcalcifications in the present study and only two (7%) lesions of clustered punctate and pleomorphic microcalcifications were detected. High rate of masses discovered in our series may be related to the routine breast US screening of patients showing dense breast composition on mammography in our unit.

On US, SA may appear as irregularly marginated masses with or without posterior acoustic shadowing and also oval or lobulated masses with well-circumscribed margins [5,16]. SA was detected as four indistinctly marginated and 1 spiculated masses on US in our series. Histologically the lesions produced ill-defined masses of firm fibrous tissue and central, dense fibrous core surrounded by softer lobulated areas. Focal acoustic shadowing without mass configuration is considered to be suspicious for malignancy [17]. Although it can be seen also in other benign breast lesions, there is little information concerning its association with adenosis in the published literature [5]. Focal acoustic shadowing without mass configuration was reported in SA very rarely with rates of 6.9% by Bilgen et al and 4.9% by Taskın et al [4,5]. We deteıted focal acoustic shadowing without mass configuration in nearly 15% of SA lesions. Histologically epithelial and myoepithelial proliferation accompanied by pronounced fibrosis was observed in these lesions.

Blunt duct adenosis is a term used to denote a lobular configuration of distended terminal ducts that have a columnar epithelium lining the central extracellular lumen and a normal lining of myoepithelial cells adjacent to the basement membrane area. Calcium phosphate microcalcifications are often found clustered in these distended ducts [18]. Mammographically, BDA is characterized by small, oval, round cluster or granular microcalcifications [19]. Barnard et al. evaluated the non-palpable mammographic abnormalities which was reported as benign at biopsy and stated that microcalcifications were mostly related with BDA [20]. In our series; 23% of BDA lesions were detected on mammography as clustered punctate microcalcifications. Additionally 69% of BDA lesions were seen on US as masses with circumscribed or non-circumscribed margins. Focal acoustic shadowing without mass configuration was detected in one (8%) BDA lesion. To the best of our knowledge, this paper is one of the few reports in English literature about sonographic appearances of blunt duct adenosis and nodular adenosis. Also this is the first study describing focal acoustic shadowing without mass configuration in BDA. Our tendency to perform routine US examination in patients that show dense mammography pattern led us to discover more frequent BDA and other subgroups of adenosis lesions on US.

Microglandular adenosis, also known as microglandular hyperplasia, is a rare variant of adenosis. It is a proliferative glandular lesion of the breast that may mimic well differentiated carcinoma both clinically and histologically [21,22]. Although MGA may be presented as a palpable mass or thickening, it is usually encountered as an incidental finding in biopsies performed for other lesions. MGA may show increased densities or calcifications that may appear suspicious for malignancy on mammography [23]. In our series MGA lesions were detected on screening imaging. MGA was detected as a spiculated mass on both mammography and US in one patient. The other two MGA lesions were seen as a microlobulated mass and an indistinctly marginated mass on US.

Adenosis was not considered as a premalignant lesion, however it was found to be associated with breast cancer especially in the forms of sclerosing and microglandular adenosis [11,22,23]. Jensen et al. and Visscher et al pointed that SA conveys an approximately doubling increase of breast cancer risk [24,25]. When atypical hyperplasia was present, this risk was raised markedly to 6.7 times [24]. SA lesions were reported to be associated with malignancy at rates ranging between 5.3% and 19% which may be observed as carcinoma or DCIS [4,12]. Likewise histopathological evaluation revealed DCIS in one lesion (3.8%) and atypical hyperplasia accompanied SA in three lesions (11.5%) in our series. Shaaban et al reported that BDA was significantly more common in cases progressing to breast cancer compared to controls and stated that related features such as atypical columnar metaplasia or atypical ductal hyperplasia may be the precursors of malignancy [26]. However columnar cell changes with atypia or atypical hyperplasia was not accompanied to BDA in our series and malignant changes were not observed in any case during follow-up (mean 36.7 months) period. Additionally association between MGA and breast cancer development has been emphasized in several studies that MGA may evolve into malignancy with higher frequency than the other forms of adenosis and nearly one third of cases of microglandular adenosis may harbour an invasive carcinoma [22,23,27,28]. Total excision has been recommended for definitive diagnosis and treatment if MGA was detected in core needle biopsy [21,23,29,30]. In our series, all MGA lesions had suspicious radiological findings for malignancy and lesions were surgically excised completely. There was no recurrence or development of carcinoma in these patients on a mean follow up of 66.3 months.

The selection of biopsy method is important for the diagnosis of adenosis lesions. Numerous studies have discussed the limitations in evaluation of breast adenosis with fine needle aspiration cytology (FNAC), core needle biopsy or frozen section [21,23,31,32,33,34,35,36]. In the series reported by Neilsen, three patients had undergone unnecessary mastectomy because of incorrectly diagnosed adenosis tumors [1]. Again Tinnemans et al described two patients in whom mastectomy was performed due to interpretation of SA as carcinoma on frozen section [33]. It may be difficult to distinguish SA from other benign or malignant lesions with fine needle aspiration cytology. It was accepted as a potential pitfall in fine needle aspiration cytology which may lead to a false-positive diagnosis [34]. Also core needle biopsy may lead to inadequate results, Westened and Liem reported false-negative core biopsy diagnosis with missed associated DCIS and invasive carcinoma in a case that only SA were identified at core biopsy [31]. Gill et al. reported that 14% of malignancies coexistent with SA were missed at core biopsy and diagnosed with excision [12]. A small incidental focus of DCIS (%10) in nodular adenosis lesion was found at excisional biopsy in the study of Dipiro et al [6]. In our series; DCIS associated with SA was not detected on core biopsy, likewise the 2 lesions with atypical hyperplasia. They were diagnosed only with surgical excisional biopsy. Only 1 atypical hyperplasia could be documented with core biopsy. FNAC may be the first-line pathological investigation in lesions categorized as BIRADS 3. Core biopsy application is needed in lesions categorized as BIRADS 4 and 5. Also suspicious FNAC findings and lesions where radiology cannot guarantee the absence of stromal invasion must be evaluated with core biopsy [35]. Excisional biopsy is required even if benign results were observed on core biopsy of BIRADS 4 and 5 lesions.

Radiological findings of adenosis can be quite similar to other benign or malignant lesions of the breast. In our series mammography was normal in 54% of SA group and most lesions were seen on US. Most frequent abnormal findings of SA were architectural distortion on mammography and masses with non-circumscribed margins and focal acoustic shadowing without mas configuration on US. In BDA usually circumscribed masses were detected on US and clustered punctate microcalcifications or circumscribed masses were seen on mammography. In microglandular type, all masses showed non-circumscribed margins (spiculated, microlobulated or indistinctly marginated) on US or mammography. In overall, majority of adenosis lesions (65%) showed suggestive radiological features for malignancy in our series. All MGA lesions displayed malignant radiological signs and percentages of lesions that were presented with malignant features for SA and BDA were 67% and 54%, respectively.

In conclusion, different types of adenosis may also become visible with malignant lesion characteristics in addition to benign appearance based on radiological findings. Especially sclerosing and microglandular adenosis may be presented with malignant features. Adenosis should be considered in order to determine the correct diagnostic approach and to avoid unnecessary mastectomy or inadequate treatment. Core needle biopsies may be the first choice for diagnosis of adenosis lesions. However if malignancy cannot be excluded, such as atypical cells are reported or presence of suspicious radiological findings besides benign core needle biopsy results, excisional biopsy has to be considered.

## Competing Interests

The authors declare that they have no competing interests.
